# Vital Hepatic Lymphoma Residuum or Excessive Immune Response? Challenging Treatment Decisions in a Patient With Systemic Lupus Erythematosus and Liver-Dominant Diffuse Large B-Cell Lymphoma: Case Report

**DOI:** 10.3389/fonc.2021.798757

**Published:** 2022-01-18

**Authors:** Lars Kurch, Thomas W. Georgi, Astrid Monecke, Daniel Seehofer, Gudrun Borte, Osama Sabri, Regine Kluge, Simone Heyn, Matthias Pierer, Uwe Platzbecker, Sabine Kayser

**Affiliations:** ^1^ Department of Nuclear Medicine, University Hospital Leipzig, Leipzig, Germany; ^2^ Department of Pathology, University Hospital Leipzig, Leipzig, Germany; ^3^ Department of Visceral, Transplant, Thoracic, and Vascular Surgery, University Hospital Leipzig, Leipzig, Germany; ^4^ Department of Radiology, University Hospital Leipzig, Leipzig, Germany; ^5^ Department of Hematology, Cellular Therapy and Hemostaseology, University Hospital Leipzig, Leipzig, Germany; ^6^ Department of Rheumatology, University Hospital Leipzig, Leipzig, Germany; ^7^ NCT Trial Center, National Center of Tumor Diseases, German Cancer Research Center (DKFZ), Heidelberg, Germany

**Keywords:** DLBCL, 18F-FDG-PET/CT, immunoresponse, systemic lupus eryhematosus, interdisciplinary approach

## Abstract

A 28-year-old female patient with active and difficult-to-treat systemic lupus erythematosus (SLE) was diagnosed with liver-dominant diffused large B-cell lymphoma. Repeated response ^18^F-FDG-PET studies showed persistently high, and, despite intensified immunochemotherapy, further increasing metabolic activity of one of the hepatic lymphoma residuals, whereas all other initial lymphoma manifestations had achieved complete metabolic remission. As biopsy of the ^18^F-FDG-PET-positive liver residual turned out to be inconclusive, complete resection was performed. Subsequent histopathological examination, however, revealed only necrotic tissue. Thus, no further lymphoma treatment was scheduled. The patient undergoes regular surveillance and is disease-free 13 months after resection. Similarly, treatment of SLE is no longer required due to lack of activity already after the first two cycles of lymphoma treatment. The case shows how closely SLE and diffused large B-cell lymphoma can be connected and stresses the importance of interdisciplinary treatment approaches. In the future, artificial intelligence may help to further classify ^18^F-FDG-PET-positive lymphoma residuals. This could lead to an increase of the positive predictive value of interim- and end-of-treatment ^18^F-FDG-PET. The patient’s point of view enables another instructive perspective on the course of treatment, which often remains hidden to treating physicians due to lack of time in clinical routine.

## Introduction

Diffused large B-cell lymphoma (DLBCL) is a highly heterogeneous disease and represents the most common subtype of non-Hodgkin’s lymphoma (NHL) in adult patients (1). Systemic lupus erythematosus (SLE) increases the risk of NHL by fourfold mainly attributed to the chronic inflammatory state, dysregulation of cytokines, and higher expression of a proliferation-inducing ligand (2). The main connecting feature of both diseases is that the B cells are at the center of the respective pathophysiological changes.

Ongoing research in DLBCL focuses on the progress of unravelling the pathogenesis and discovering of new molecular markers, thus providing the background for molecularly targeted strategies (3, 4). In routine clinical practice, the International Prognostic Index (IPI) and its adaptions, e.g., Central Nervous System (CNS)-IPI, are used to estimate prognosis at diagnosis (5, 6). Standard first-line immunochemotherapy consists of rituximab, cyclophosphamide, doxorubicin, vincristine, and prednisone (R-CHOP). About two-thirds of DLBCL patients achieve long-term disease-free survival by R-CHOP, whereas the other one-third have a poor prognosis and low response rates to salvage treatment (7).

Morphologic response is routinely assessed by computed tomography (CT) scans. Disappearance of lymphoma lesions is termed as complete response (CR) and regression of at least 50% as partial response (PR). However, CR is rarely achieved, and lymphoma residuals representing PR may either be vital or avital lymphoma tissue.

Metabolic imaging by positron emission tomography (PET) using the radiotracer 18-fluorodeoxyglucose (^18^F-FDG) is capable of further distinguishing residual tumor tissue based on metabolic response. The Deauville score (DS) is used to graduate metabolic response in interim and end-of-treatment ^18^F-FDG PET scans. It comprises five categories which are defined as scores 1 (no residual uptake), 2 (residual uptake ≤ mediastinal uptake), 3 (residual uptake > mediastinal uptake, but ≤ liver uptake), 4 (residual uptake > liver), and 5 (residual uptake >> liver). Since the DS is ordinal and prone to interobserver variability, quantitative measurements, e.g., maximum standard uptake values (SUV_max_) or qPET (the quotient of mean SUV of the four hottest connected voxels within lymphoma residual and mean SUV measured within a 30-ml volume of normal liver parenchyma) are used in addition (8, 9). Currently, lymphoma residuals with DS 1 to 3 are considered complete metabolic response (9). Using this cutoff ^18^F-FDG PET yields a negative predictive value (NPV) of >80% already after the second cycle of R-CHOP (7). The positive predictive value (PPV), however, ranges between 30% and 40%. It may increase up to 70% if also lymphoma residuals showing a DS of 4 are regarded as complete metabolic response (7). Of note, shifting the cutoff accordingly does not result in a significant drop of the NPV (7, 8). Nevertheless, false-positive findings may occur, are challenging for the treating physicians, and may lead to unconventional treatment decisions, as in our patient.

## Case Description

In October 2018, a 28-year-old female patient presented to her local hospital with musculoskeletal complaints and pericardial effusion. Antiphospholipid syndrome (proven positivity for anticardiolipin and anti-β2-glucoprotein antibodies as well as for lupus anticoagulant) was diagnosed in 2011 after deep pelvic venous thrombosis with consecutive central pulmonary embolism had occurred. In the context of antiphospholipid syndrome, the patient suffered an embolic stroke in 2014, since phenprocoumon was mistakenly discontinued.

On suspicion of incipient SLE, treatment was started with colchicine (0.5 mg/2nd day) and a nonsteroidal anti-inflammatory drug. For further diagnosis, the patient presented to our rheumatology outpatient clinic in December 2018. Laboratory examination revealed high antinuclear antibody titer (1:5120; normal: <1:80), positive antidouble-stranded DNA (57.1 IU/l; normal: <20), anti-Smith, antiphospholipid (anticardiolipin 118.1 U/ml, normal: <7; anti-β2-glykoprotein 86.4 U/ml, normal: <5), and nucleosome antibodies (>200 U/ml, normal: <20) as well as low C4 level (0.09 g/l, normal range: 0.15–0.43). Thus, the diagnosis of SLE was made based on clinical findings and serological markers in accordance to the Systemic Lupus Collaborating Clinics (SLICC) criteria (10). Colchicine was left unchanged and hydroxychloroquine was added (200 mg / day). Despite this treatment, the patient developed pericarditis, pleuritis, and pancytopenia in June 2019. Colchicine was stopped, hydrochloroquine dose was increased to 400 mg/day and azathioprine 75 mg/day was added. Prednisone was started at 250 mg/day for 3 days and thereafter continued at 70 mg/day with consecutive tapering. As the platelet count did not rise, azathioprine was replaced after 2 months by mycophenolate mofetil at 1,000 mg/day. However, platelet count dropped further, minimally to 3 × 10^9^/l (normal range: 150–400 × 10^9^/l). Consequently, in January 2020, hydroxychloroquine and mycophenolate mofetil were stopped and intravenous immunoglobulin therapy (2 × 30 g) was given. Due to lack of response, treatment was further escalated (February 2020) using rituximab 1,000 mg once monthly and prednisone 60 mg/day with consecutive tapering.

**Figure 1:**
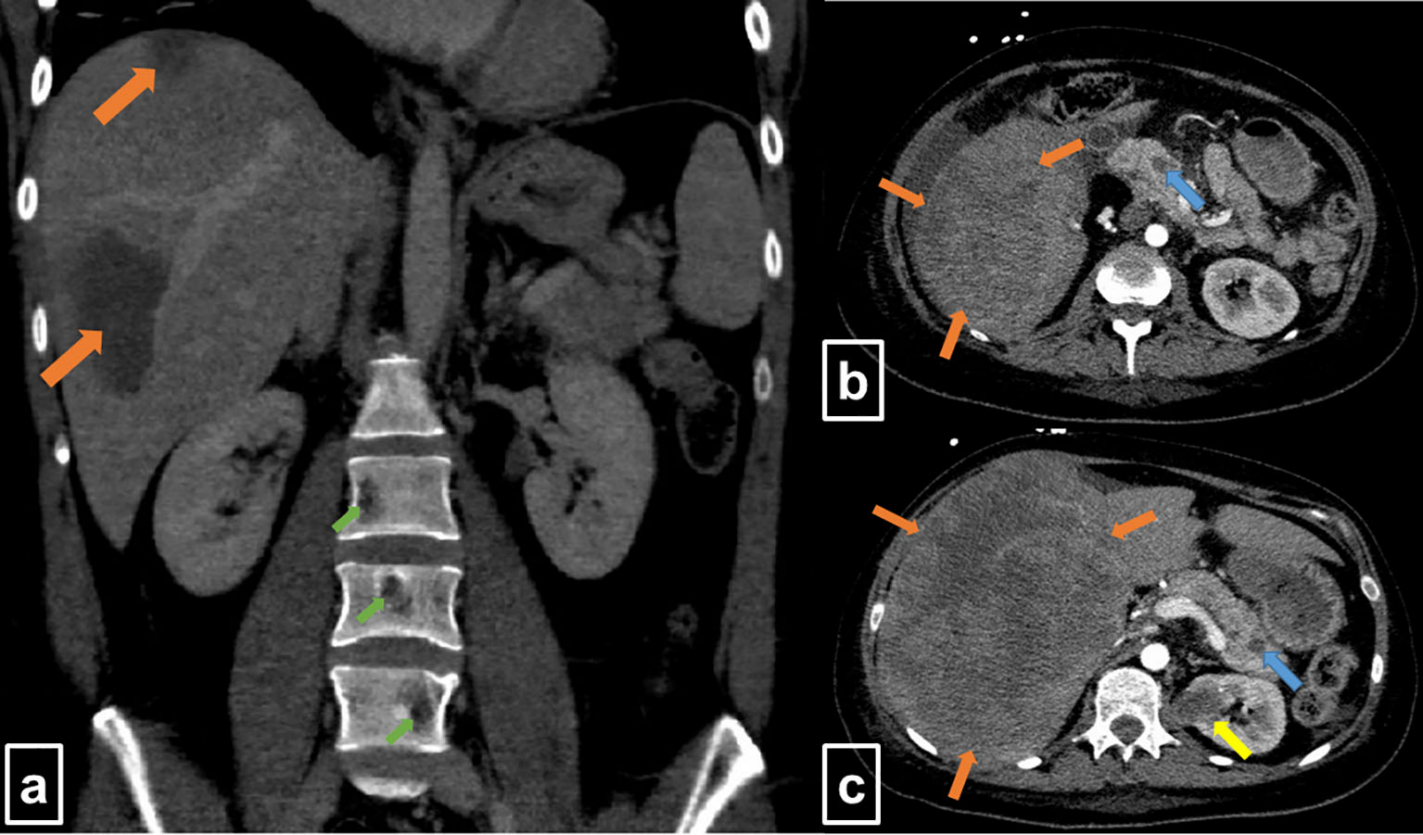
Contrast-enhanced computed tomography at initial staging (Mar 2020). **(A)** The coronal slice shows two large hypodense lesions in the right liver lobe (orange arrows) and osteolytic lesions of lumbar vertebrae (green arrows). **(B, C)** Transversal slices display extended, hypointense lesions in the liver (orange arrows), as well as lesions in the pancreas (blue arrows) and the left kidney (yellow arrow).

On hospital admission for the second cycle of rituximab, the patient complained of pain in the right upper abdomen. Ultrasound revealed extensive tumor masses in the liver. As the patient reported of resolved hepatitis B during childhood, hepatocellular carcinoma was the main differential diagnosis. Computed tomography (CT) covering neck, thorax, abdomen, and pelvis confirmed large tumor masses in the right liver lobe of 10.9 × 9.7 × 13.6 and 14 × 13 × 16 cm (719 and 1,456 ml), compatible with hepatocellular carcinoma (**Figures 1A–C**). In addition, metastases were suspected in the left supradiaphragmatic recess, in both kidneys, pancreas, and skeleton (**Figures 1A–C**). However, core needle biopsy of the liver masses revealed in parts necrotic decomposed DLBCL. Immunohistochemistry was positive for CD20, multiple myeloma antigen 1, and B-cell lymphoma (BCL) 6 but negative for BCL2, CD10, CD30, and latent membrane protein 1. Epstein-Barr virus (EBV)-encoded small RNA *in situ* hybridization and c-MYC colorimetric *in situ* hybridization were negative. Thus, nongerminal center, nondouble-hit, EBV-negative DLBCL was diagnosed by the end of March 2020. Cerebrospinal fluid puncture revealed no malignant cells. Taken together, the patient had an Ann Arbor stage of IVA. IPI was 4 and CNS-IPI was 5 (ECOG performance status of 2; increased lactate dehydrogenase; stage IV disease; ≥1 extranodal lesion, kidney involvement), suggesting a high-risk constellation as well as an increased risk for CNS relapse (6).

Treatment consisted of standard immunochemotherapy with six cycles of R-CHOP alternated with two cycles of high-dose methotrexate (HD-MTX, 3g/m²) in combination with rituximab (R-HD-MTX) after cycles two and five of the R-CHOP therapy.

To prevent reactivation of hepatitis B during immuno-suppressive chemotherapy, tenofovir was given (11).

**Figure 2:**
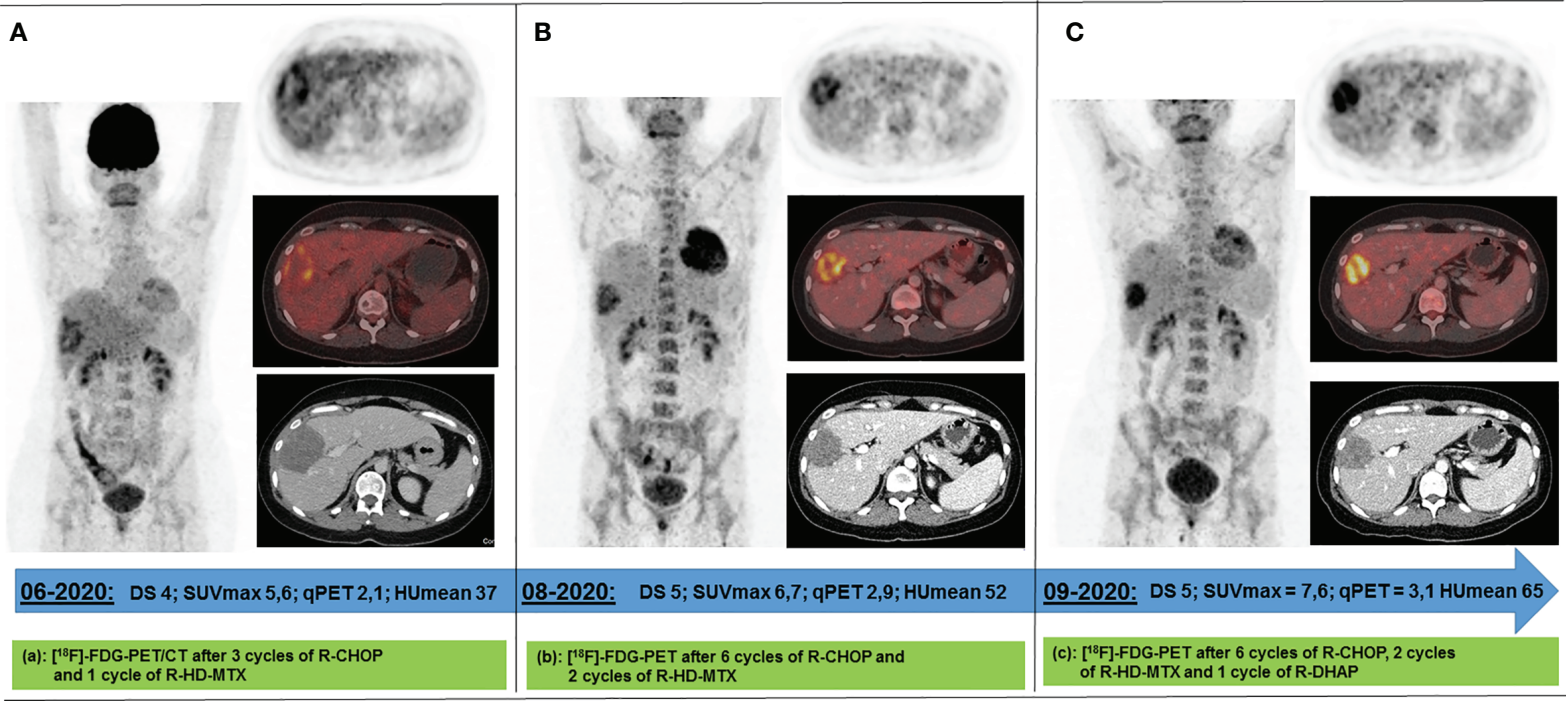
Response ^18^F-FDG PET scans acquired between Jun 2020 and Sep 2020 following 4 **(A)**, 8 **(B)**, and 9 **(C)** cycles of immunochemotherapy. The only remaining metabolically active lesion after 4 immune-chemotherapy cycles has been a circumscribed area in the right liver lobe. Under continued **(B)** and intensified immunochemotherapy **(C)**, its metabolism increased while its volume decreased. DS, Deauville score; SUV_max_, maximum standard uptake value; qPET, quantitative positron emission tomography; HU_mean_, average value of Hounsfield Units.

For interim response evaluation, ^18^F-FDG-PET/CT was performed after three cycles R-CHOP and one cycle R-HD-MTX (June 2020), revealing residual metabolic activity in one of the two declined liver lesions (DS4, qPET 2.11; 7.0 × 5.4 × 8.0 cm = 151 ml) (**Figure 2A**). For all other lesions ^18^F-FDG/PET showed a complete metabolic response (DS 1). Consistently, CT scan revealed a morphologic PR.

**Figure 3:**
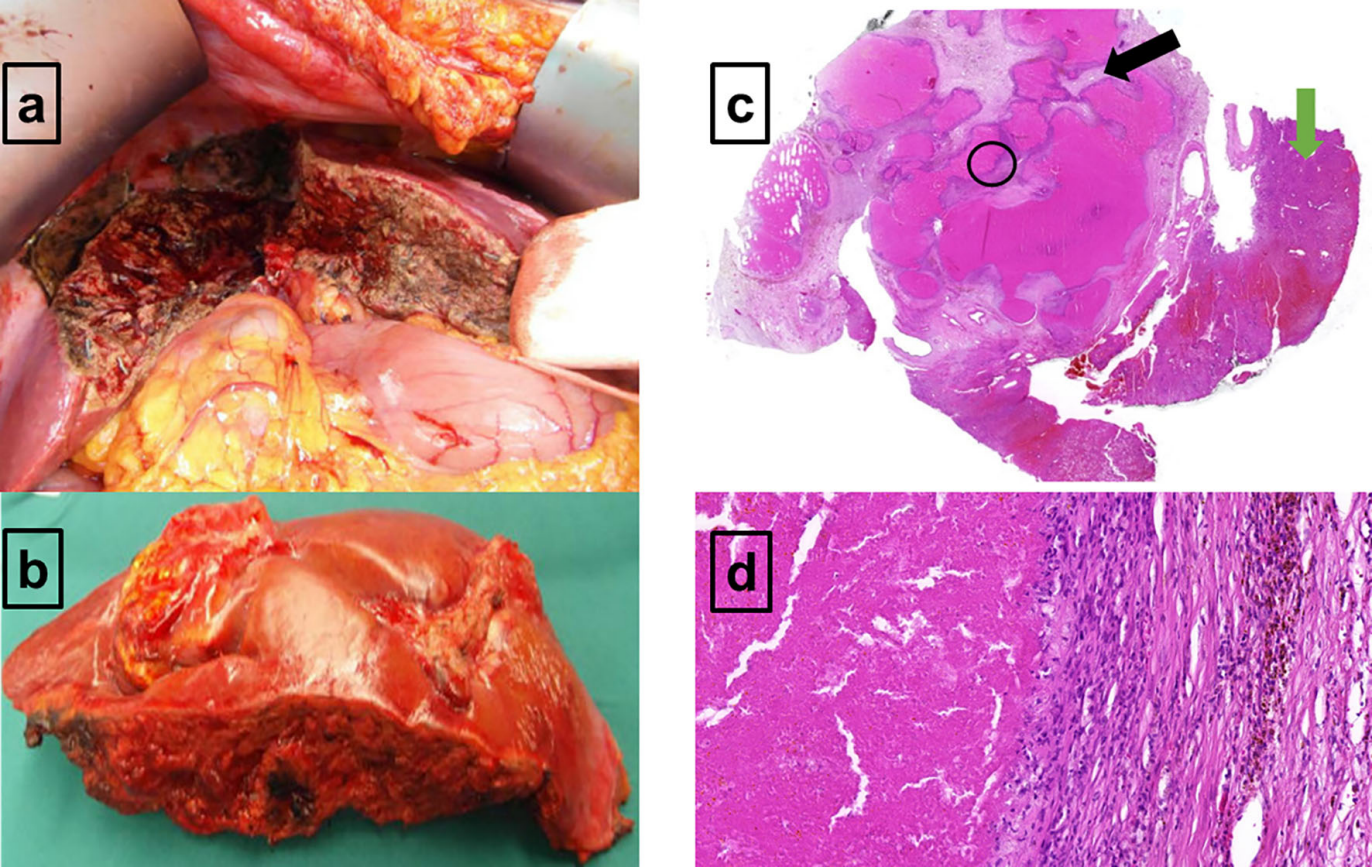
Macroscopic and microscopic presentation of the ^18^F-FDG-PET-positive residual in the right liver lobe **(A–D)**. **(A)** The *in situ* view demonstrates the liver segments V and VIII which are mostly necrotic and remodeled. **(B)** Macroscopic resectate of liver segments V and VIII. **(C)** Microscopic view of the liver resectate shows normal liver parenchyma on the right (green arrow) and extended area of necrosis on the left (black arrow). No evidence of large, polymorphous lymphoma cells. **(D)** Granulation zone with macrophages, small lymphocytes, and hemorrhagic remnants on the right, and an area of necrosis on the left (**D** corresponds to a close-up of the black circled area visible on **C**). No evidence of large, polymorphous lymphoma cells.

After completion of the immunochemotherapy, a second response ^18^F-FDG-PET/CT evaluation was performed (August 2020). The liver lesion had further decreased (4.8 × 4.8 × 6.9 cm = 79 ml), whereas its metabolism had increased (DS5, qPET 2.87) (**Figure 2B**). Since refractory disease seemed likely, one cycle of standard salvage treatment with rituximab, dexamethasone, cytarabine, and cisplatin (R-DHAP) was administered at the beginning of September 2020 (12). Fourteen days later, autologous stem cells were harvested (9.03 × 10^6^ CD34+ cells/kg body weight). ^18^F-FDG-PET/CT was repeated at end of September 2020. The images showed that the liver lesion had slightly decreased in terms of morphology (4.6 × 4.0 × 6.0 cm = 55 ml), but its metabolism had further increased (DS5, qPET 3.11) (**Figure 2C**). For histopathology analysis, an ultrasound-guided core needle biopsy was performed. However, histopathological analysis was inconclusive. Various potential treatment options were discussed with the patient, including surgical resection of the active liver residual, autologous and allogeneic hematopoietic stem cell transplantation, as well as chimeric antigen receptor (CAR) T-cell therapy. In October 2020, the interdisciplinary board decided to have the liver segments containing the ^18^F-FDG-PET-positive lesion removed. Histopathology and immune-histochemical analyses of the removed liver segments (V/VIII) revealed only necrosis surrounded by numerous macrophages and small lymphocytes without any evidence of lymphoma cells (**Figure 3**). Nested polymerase chain reaction on DNA from hepatitis B virus was also negative.

The patient did not receive any further treatment for DLBCL and underwent routine surveillance. An ^18^F-FDG-PET/CT scan performed mid of April 2021 revealed metabolic CR. The patient is disease free also 13 months after resection (last update: November 2021).

Of note, all SLE-related complaints had already resolved after the first two cycles of R-CHOP. Thus, also prednisone was terminated in July 2020 in agreement with the rheumatologists, who were regularly consulted during the patient’s lymphoma therapy. Also, when the PET-positive liver residual became more active (August/September 2020), no SLE-related symptoms were reported by the patient.

The patient was recently interviewed on her view concerning the disease and treatment approach. She described herself as being tough, strong minded, positive thinking, and future oriented. At the time of her DLBLC diagnosis, she was already very alarmed since the treatment of SLE was highly complicated. The lymphoma diagnosis had caused her further worries, but her familiar background (husband and her daughter of eight years) stimulated her to fight. Her optimism grew already after the first chemotherapy cycle, as the SLE-related symptoms had rapidly decreased, and she had felt constantly better. Despite the residual finding in the liver, she was very pleased with the results of the first ^18^F-FDG/PET, as all other lesions showed no activity. She even remained confident after the second ^18^F-FDG/PET scan continued to show the active focus in the liver, knowing that there were still many treatment options. Nevertheless, regarding the CAR T-cell approach, she felt reluctant considering cost/effectiveness, though her health insurance would have fully covered the costs for this approach. After the third ^18^F-FDG/PET examination still showed activity of the liver residual despite intensified therapy, she was deeply worried. However, the option to have the unclear and maybe active lymphoma in the liver completely resected, was relieving for her. Therefore, she was immediately eager to have the surgery done. After the histopathological analysis of the resected tissue confirmed absence of DLBCL, she felt a great sense of gratitude and relief. Currently, the patient is working again, feels healthy, and is confident about her future. When asked about the resources that helped her during the course of the disease, she mentioned the competent medical and psycho-oncological care, her family, her network of friends, her two dogs, and the experience of having coped with previous serious illnesses already as a young woman.

## Discussion

As SLE increases the risk of NHL by fourfold (2), it seems tempting to speculate that the DLBCL arose secondary to the chronic inflammatory disease. Indeed, the presented case suggests a possible link between autoimmune reactive diseases as an underlying chronic inflammatory trigger factor for NHL, a phenomenon that is particularly known in extranodal marginal zone lymphoma of mucosa-associated lymphoid tissue lymphomas (13). Chronic B-cell stimulation and antigenic drive, a hallmark in autoimmune disorders such as rheumatoid arthritis, SLE, and celiac disease may play important roles in autoimmunity-related lymphomagenesis (14).

Aside from speculation, CT scan at diagnosis revealed a relatively uncommon pattern of DLBCL involvement with lesions in the liver, pancreas, kidneys, and skeleton, but only one affected lymph node region. Based on a large cohort of roughly 26,000 adult DLBCL patients, Castillo et al. found dissemination pattern not including nodal sites of head/neck but liver and/or pancreas to be associated with significantly worse outcome (15). Correspondingly, IPI and CNS-IPI suggested a high-risk constellation, also for CNS relapse (6). In general, patients with DLBCL have a 2%–5% CNS relapse rate after therapy with R-CHOP. In our patient, CNS relapse rate was augmented to 10% due to a CNS-IPI of 5 (6, 16). Although data on CNS recurrence prophylaxis lack robust evidence concerning patient selection criteria, optimal time-points, and ways of administration (17), we decided on intravenous high-dose R-HD-MTX following 2nd and 5th cycles of R-CHOP. This procedure was in line with the recommendations of the British Society for Haematology (17). Currently, it is a matter of debate if the route of administration has an impact on the CNS relapse rate. In a large retrospective cohort analysis, Orellana-Noia et al. found no significant difference in CNS relapse rates between intrathecal or systemic high-dose methotrexate administration (18).

Following three cycles R-CHOP and one R-HD-MTX, all lesions except one in the liver were negative on ^18^F-FDG-PET scan. Since this lesion had decreased compared with baseline, immunochemotherapy with R-CHOP was continued. However, after six cycles of R-CHOP and two cycles of R-HD-MTX, ^18^F-FDG-PET scan showed further increased metabolism of the liver residual as compared with the previous ^18^F-FDG-PET scan. In quantitative terms, the qPET value had risen from 2.1 to 2.9. In interim ^18^F-FDG-PET following two courses of R-CHOP such a high qPET value corresponds to a PPV of 60% (8). In an end-of-treatment ^18^F-FDG-PET scan, however, the respective PPV is expected to be even higher, rendering refractory disease more likely. At this point, obtaining a biopsy from the ^18^F-FDG-PET-positive liver lesion would have been an alternative before administering R-DHAP. After one cycle R-DHAP size of the residual liver lesion had slightly decreased, whereas metabolism as measured by ^18^F-FDG-PET had again increased. A subsequently performed ultrasound-guided core biopsy was inconclusive, which may occur in roughly 8% as compared with about 3% in the case of an excisional biopsy (19). Thus, surgery of the ^18^F-FDG-PET-positive liver residual appeared to be the best option to receive reliable histopathology results to guide further treatment decisions (20, 21). In case of persistent DLBCL CAR T-cell therapy in patients with primary refractory disease after at least two lines of therapy would have been an option (22). In those patients with achievement of CR after further intensive chemotherapy treatment (e.g. rituximab, ifosfamide, carboplatin, and etoposide) autologous stem cell transplantation would have been a reasonable approach. Currently, biomarker-driven salvage regimens, incorporating, e.g., ibrutinib for relapsed and refractory DLBCL, are under clinical investigation (23, 24).

As no lymphoma cells were detected within the resectate by detailed histopathology analysis, our patient continued with regular follow-up examinations.

Using the most widely available and intensively studied radiotracer ^18^F-FDG to evaluate response to lymphoma treatment yields a certain number of false-positive findings due to the lack of the tracer to distinguish inflammation from vital tumor tissue (25).

False-positive findings on ^18^F-FDG-PET images may especially occur if the scan is performed shortly after the last chemotherapy cycle. However, since each ^18^F-FDG-PET scan was performed earliest 14 days after the last chemotherapy administration, premature scanning can be excluded as a reason for false-positive findings (26). Another reason for false-positive findings are new lesions which occur especially after immunosuppressive chemotherapy. They are often located pulmonary or in cervical, hilar, and inguinal lymph nodes. Provided that all initial lymphoma manifestations show significant reduction in its activity or a normalized metabolism, these new lesions are usually inflammatory (25, 27). However, in the presented case, the metabolically increasing liver lesion corresponded to an initial lymphoma manifestation.

Inflammatory immune reactions as a further reason for false-positive findings in ^18^F-FDG-PET are especially triggered by large and in parts necrotic decayed lymphoma masses as well as through cell death induction by rituximab containing immunochemotherapy (28, 29). Macrophages play an important role for clearance of cell and tissue debris. In addition to macrophages migrating from the blood into decaying lymphoma tissue, the liver is densely equipped with stationary macrophages. The interaction between migrated and stationary macrophages could have resulted in a strong local immune response. This hypothesis is supported by the fact that numerous macrophages were found in the periphery of the ^18^F-FDG-PET-positive lesion. Furthermore, aberrations of macrophage phenotype and function could be demonstrated in patients with autoimmune diseases (29). As one consequence, the balance between pro- and anti-inflammatory operating macrophages is shifted towards proinflammation (29). Thus, in our patient suffering from active SLE, a shift of macrophages activity towards inflammation could also be a reasonable explanation for the increased glucose metabolism in an otherwise avital lymphoma residual.

To increase the PPV of interim- and end-of-treatment ^18^F-FDG-PET, it is necessary to further discriminate ^18^F-FDG-PET-positive lymphoma residuals. For this, the application of artificial intelligence (AI) could be promising (30). Currently, AI is particularly applied to automatically extract all lymphoma lesions from the initial ^18^F-FDG-PET scan to characterize them more precisely and to calculate the total tumor volume in a convenient way (31). However, to perform more sufficient and reliable assessments of metabolically active lymphoma residues, it seems necessary that a machine-learning system is trained with information from a large number of patients. Based on the case presented here, it could be important to consider clinical and paraclinical information (e.g., presence of acute and chronic inflammatory diseases; degree of activity of inflammatory diseases and its development during the course of treatment; histopathological features of the lymphoma; administered chemotherapeutic agents; results of biopsies taken from active residuals; survival data) as well as various image-based parameter (e.g., initial lesion volume and its dynamics during the course of treatment; dynamics of Hounsfield Units, SUV, and qPET values). As datasets of a large number of patients are required to optimally train such a system, international cooperation becomes crucial. An example for such a cooperation is the PETRA consortium located in Amsterdam (7).

In conclusion, false-positive lymphoma residuals on ^18^F-FDG-PET scans may complicate treatment decisions. In case of a singular ^18^F-FDG-PET-positive lymphoma residual at the end of standard immunochemotherapy, entire resection and histopathological examination yields important information to decide on the best treatment option. In the future, however, artificial intelligence could be helpful in assessing ^18^F-FDG-PET-positive lymphoma residuals in a more differentiated manner since many parameters from the residual as well as clinical information could be considered at the same time.

## Data Availability Statement

The raw data supporting the conclusions of this article will be made available by the authors upon reasonable request.

## Ethics Statement

Ethical review and approval was not required for this case report. This is in accordance with the local legislation and institutional requirements. Patients declare on their admission to the University Hospital of Leipzig that their data can be used in anonymized form for scientific evaluation and for publication. Concerning this case report, the local data safety commissioner did not identify any kind of data safety violation.

## Author Contributions

LK, TG, and SK were responsible for the concept of this paper, contributed to the literature search and data collection, analysed and interpreted data, and wrote the manuscript. SH and MP treated the patient. AM, DS, GB, OS, and RK performed research and critically revised the manuscript. UP critically revised the manuscript. All authors contributed to the article and approved the submitted version.

## Funding

Open Access funding enabled and organized by Projekt DEAL.

## Conflict of Interest

The authors declare that the research was conducted in the absence of any commercial or financial relationships that could be construed as a potential conflict of interest.

## Publisher’s Note

All claims expressed in this article are solely those of the authors and do not necessarily represent those of their affiliated organizations, or those of the publisher, the editors and the reviewers. Any product that may be evaluated in this article, or claim that may be made by its manufacturer, is not guaranteed or endorsed by the publisher.
